# Prevention through Activity in Kindergarten Trial (PAKT): A cluster randomised controlled trial to assess the effects of an activity intervention in preschool children

**DOI:** 10.1186/1471-2458-10-410

**Published:** 2010-07-12

**Authors:** Kristina Roth, Sonja Mauer, Matthias Obinger, Katharina C Ruf, Christine Graf, Susi Kriemler, Dorothea Lenz, Walter Lehmacher, Helge Hebestreit

**Affiliations:** 1 University Children's Hospital, Julius-Maximilians-University Wuerzburg, Josef-Schneider-Str. 2, 97080 Wuerzburg, Germany; 2 Institute of Sports and Sport Science, Julius-Maximilians-University Wuerzburg, Judenbuehlweg 11, 97082 Wuerzburg, Germany; 3 Institute of Theory and Practice of Training and Movement, German Sport University Cologne, Am Sportpark Muengersdorf 6, 50933 Cologne, Germany; 4 Institute of Exercise and Health Sciences, University of Basel, St. Jakob-Turm, Birsstr. 320b, 4052 Basel, Switzerland, and Swiss Tropical and Public Health Institute, Socinstr. 57, P.O. Box, 4002 Basel, Switzerland; 5 Institute of Medical Statistics, Informatics and Epidemiology, University of Cologne, 50924 Cologne, Germany

## Abstract

**Background:**

Physical activity and motor skills acquisition are of high importance for health-related prevention and a normal development in childhood. However, few intervention studies exist in preschool children focussing on an increase in physical activity and motor skills. Proof of positive effects is available but not consistent.

**Methods/Design:**

The design, curriculum, and evaluation strategy of a cluster randomised intervention study in preschool children are described in this manuscript. In the Prevention through Activity in Kindergarten Trial (PAKT), 41 of 131 kindergartens of Wuerzburg and Kitzingen, Germany, were randomised into an intervention and a control group by a random number table stratified for the location of the kindergarten in an urban (more than 20.000 inhabitants) or rural area. The aims of the intervention were to increase physical activity and motor skills in the participating children, and to reduce health risk factors as well as media use. The intervention was designed to involve children, parents and teachers, and lasted one academic year. It contained daily 30-min sessions of physical education in kindergarten based on a holistic pedagogic approach termed the "early psychomotor education". The sessions were instructed by kindergarten teachers under regular supervision by the research team. Parents were actively involved by physical activity homework cards. The kindergarten teachers were trained in workshops and during the supervision. Assessments were performed at baseline, 3-5 months into the intervention, at the end of the intervention and 2-4 months after the intervention. The primary outcomes of the study are increases in physical activity (accelerometry) and in motor skills performance (composite score of obstacle course, standing long jump, balancing on one foot, jumping sidewise to and fro) between baseline and the two assessments during the intervention. Secondary outcomes include decreases in body adiposity (BMI, skin folds), media use (questionnaire), blood pressure, number of accidents and infections (questionnaire), increases in specific motor skills (throwing, balancing, complex motor performance, jumping) and in flexibility.

**Discussion:**

If this trial proofs the effectiveness of the multilevel kindergarten based physical activity intervention on preschooler's activity levels and motor skills, the programme will be distributed nationwide in Germany.

**Trial Registration:**

ClinicalTrials.gov Identifier: NCT00623844

## Background

Physical activity and motor skills acquisition play a key role in childhood development especially during the preschool period. Engaging in a variety of motor tasks stimulates the neuromotor system and enables the child to rely on a large and stable store of experiences and to adjust it to new situations [1].

Physical activity intervention programmes have been shown to improve coordinative skills in preschoolers [2] and may result in a reduction of accidents [3,4]. As current research describes a secular decline in coordinative motor skills in preschool children [5], promotion of physical activity and motor skills gain more and more importance in today's kindergartens. Moreover, a lack in physical activity has been associated with an increased risk of obesity, and physical activity can help to prevent childhood obesity [6]. Over the last years, obesity has become a major issue even in preschool children. In the United States, the number of obese 2- to 5-year-old children has risen dramatically to 26% [7]. In Germany, a representative national survey, the Kinder- und Jugend-Gesundheitssurvey (KiGGS) found that 9.3% of the 3- to 6-year-old girls and 8.9% of the 3- to 6-year-old boys were overweight or obese (body mass index >90^th ^centile) [8]. In the age group of the 7- to 10-year-olds, the respective prevalence is much higher in girls (14.7%) and boys (15.9%) [8]. Thus, increasing physical activity in preschool children might be a valuable mean to improve the children's development, reduce accidents and prevent obesity. Furthermore, an education towards a physically active lifestyle at this age could even shape long-term habits associated with future health.

Increasing physical activity and motor skills in preschool children has been the aim of several projects. However, the number of high quality randomised controlled trials in this age group is low [9] and data reported are conflicting. While some [10] but not all [11] educational interventions targeting parents, teachers and children showed no benefits, interventions which included an activity programme had some positive effects on motor skills [2-4,9] and adiposity [12]. This conclusion is in line with a recent review on school-based interventions concluding that a mandatory physical activity component will boost the effects of obesity prevention interventions [13].

The holistic pedagogic approach termed the "early psychomotor education" is one age-appropriate and promising option for an education towards an active lifestyle in preschool children and, at the same time, an improvement in motor skills [14]. The psychomotor approach considers the sensory, motor, social, emotional and cognitive development of children and, thus, is a very integrating way to assist children in their development [15]. Children are encouraged to increase their self-competence, social competence and competence in dealing with materials and contents of every-day life [16]. The theory proposes an ideal learning environment in which defined or regulated movements or rules do not exist. Therefore, activity tasks never consist of simply imitating movements but always prompt the children to creatively deal with the materials and tasks. Furthermore, movements are not evaluated and children are not judged on their movement skills but they are rather invited to find their own solutions for the tasks they are encountering. Consequently, they can engage freely and with joy in physical activity.

The main objective of this project was to develop and evaluate a child-appropriate kindergarten programme to enhance physical activity and motor skills in 4- and 5-year-old children. To achieve these goals, the intervention programme was based on the holistic pedagogic approach outlined above and was required to be easily implemented in facilities of different sizes and with different equipment. The programme comprised different elements including movement and concentration tasks, tasks based on rhythmic or musical contents, tasks that focused on acting and identifying with certain roles and motor-skills based tasks [15,16]. One additional goal of the intervention was the parallel education of children, parents and kindergarten teachers to stimulate and facilitate the continuation of the programme after the intervention without being dependent on the expertise of the study personal.

## Methods and Design

### Study Design

This cluster-randomised, controlled trial in kindergartens investigated the efficacy and feasibility of a physical activity intervention to improve physical activity and other health outcomes in 4- and 5-years-old children. The intervention was designed for one kindergarten year and targeted the participating children, their parents and their kindergarten teachers. The children received a daily physical education lesson of at least 30 min duration taught by the kindergarten teachers for one academic year. The parents were invited to educational evenings and periodically received written information. Activity homework cards were given to the families. The kindergarten teachers were equipped with instructional materials and trained during workshops. Supervisions were realised repeatedly during the intervention. Assessments of the children took place at baseline before the intervention started, twice during the intervention, and 2-4 months after the end of the intervention.

The study was approved by the Ethics Committee of the Medical Faculty of the University of Wuerzburg.

### Recruitment

Due to a reported high incidence of obesity at school entry, the region around Kitzingen and Wuerzburg, Germany, was chosen for the intervention programme [17]. All suitable 131 kindergartens in this area were invited to participate in the PAKT-Study in autumn and winter 2006/2007. Kindergartens with an existing physical activity promotion programme were not approached. 41 kindergartens volunteered to participate in the trial. All parents of children, who were 4.0 up to 5.9 years at the start of the intervention, were invited to take part in the study. Written informed consent was provided from the legal guardians for 744 of 979 eligible children. Children with chronic health problems which limited exercise capacity were not included. 709 children in the required age participated in a baseline testing in summer 2007. Thereafter, the kindergartens were randomly assigned to a control (n = 20) or an intervention group (n = 21). The randomisation was done on the level of the kindergartens, not of the children for organisational reasons. Randomisation was performed using a random number table by a researcher blinded to the identity of the kindergartens and was stratified to the location of the kindergartens (urban vs. rural). Each kindergarten was informed about the result of allocation after randomisation by a telephone call. The intervention programme started with 368 children in September 2007.

**Figure 1:**
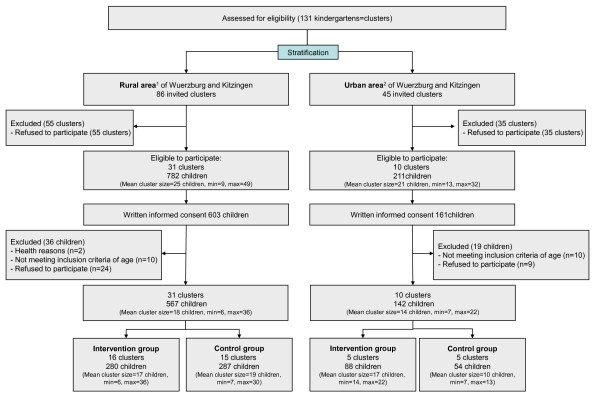
**Flow diagram of the stratification and randomisation of clusters and individuals**. 1) rural area - kindergarten is located in a village with <20,000 inhabitants. 2) urban area - kindergarten is located in a town with ≥20,000 inhabitants.

Figure 1 summarises the sample stratification for the PAKT-study.

### Sample size justification

Assuming a gender specific effect of a physical activity intervention, the statistical power had to be strong enough to allow separate analyses for girls and boys. A group difference of 0.6 standard deviations after intervention might be relevant for preventive reasons. With an usual main level of significance α = 0.025 (two primary outcomes) and β = 0.80 power, a minimum of 53 persons per group have to be available for analysis in a non-clustered design. To account for possible missing data from both follow-up visits during the intervention period due to non-participation in both tests or due to drop-out - estimated to occur in about 5% of the participating children - sample size has to be adjusted accordingly. Since accelerometry was required for one of the primary outcomes and accelerometry data are often too incomplete for valid analysis, sample size was further increased by 20%.

Because of cluster sampling (randomisation of children into intervention or control group according to the child's kindergarten) the number of subjects needed was increased by another 30% in the initial sample size calculation. In order to meet current standards of power calculation in cluster randomised trials we performed an additional calculation considering intraclass correlation. Assuming an intraclass correlation of ρ = 0.1 [18] and an average cluster size of 17 children per kindergarten, the sample size had to be increased by 160% [19].

Thus, a total of 174 girls and 174 boys were required for the intervention group and the same numbers of boys and girls for the control group.

### Intervention

The intervention, developed by physical education scientists, paediatricians, dieticians and a physiotherapist, targeted three groups: the children themselves, their parents and their kindergarten teachers.

In the intervention group, all kindergarten teams and their families received the same curriculum, training instructions and educational materials. The kindergarten teachers were trained and supervised by physical education specialists and a physiotherapist in planning and teaching the physical education lessons. The study personal also prepared the information for the parents and conducted the educational evenings.

The kindergartens in the control group continued their usual routine with one physical activity lesson per week lasting 30 to 45 minutes and realised in their own way and focus. Kindergarten teachers and parents in the control group were informed about the study design, the testing and the intervention arm but did not know any further details regarding the intervention topics and modules.

#### Daily physical activity lessons

Daily physical activity lessons lasting at least 30 min were realised in all kindergartens of the intervention group. If a lesson had to be cancelled, the missed activity time was added to the following lesson to ensure a weekly activity time of at least 2.5 hours.

A collection of games and exercise tasks, based on the psychomotor approach, was developed to help the kindergarten teachers to plan the physical activity lessons. Each game or task was described by written instructions, some of them were additionally explained with pictures. The educational contents were provided as cards, subdivided into different categories such as games for improving the coordination, power etc. These materials enabled the kindergarten teachers to plan and realise the lessons within the general predetermined structure while allowing an individual focus and acknowledging the spatial situation as well as the staff resources of the kindergarten.

The structure of the physical activity lessons was standardised as follows: An initial ritual clearly marked the start of the lesson and helped the children to join the group. After that, an introduction prepared the children for the tasks and introduced the theme or the special educational goal of the lesson. The main section of the lesson focussed on the training of perception and coordinative skills. Additionally, there were games and exercises to improve physical endurance, speed, power, creativity, flexibility, cooperation and throwing skills. At the end of each lesson, a final cool-down game was realised, and followed by a feedback round. This structure was maintained throughout all lessons. Nevertheless, ideas for games and tasks were provided in the game collection that enabled the teachers to spontaneously include the children's creativity and their playing and acting ideas into the lessons. According to the psychomotor concept, this is a strong path to enable the children to utilise their motor abilities self-confidently and autonomously during and beyond the lessons.

#### Physical activity homework cards

52 activity homework cards were created by a physiotherapist and a physical education specialist. These cards included activity games and motor tasks for a single child as well as games with a focus on team play. The latter games focussed on the active cooperation of the whole family. To overcome possible language barriers in some non-German speaking families the games and motor tasks on the homework cards were illustrated by pictures.

Every week the kindergarten teachers individually chose one or two homework cards with respect to the current season (Easter games etc.), weather (games for inside or outside play) or theme that paralleled the actual physical education topic in the kindergartens (i.e. balancing abilities). The activity homework was practiced with the children at kindergarten to encourage them to independently exercise at home together with their parents, siblings or other playmates. On the backside of each card further information was provided to the parents concerning the aim of the exercise, special needs and possibilities of adjusting the level of difficulty.

Prior to the holiday breaks, children and their families received special seasonal activity cards with games and ideas for an active family time (i.e. Christmas, Easter or Pentecost card).

#### Education of parents

The parents were invited to three educational seminars. The first seminar took place in nine different locations/kindergartens with a parental attendance rate of about 45%. The nine central and easily accessible locations were distributed in the area of the intervention. The second seminar was organised in six locations which were different from the locations of the first seminar. Here, parental attendance was 21%. As there was one kindergarten in the intervention group with a high proportion of migrants from Russia, a Russian speaking person joined the first two educational seminars for parents from this kindergarten. In retrospect, knowledge of the German language of attending parents was generally sufficient. However, the involvement of the Russian contact person facilitated the cooperation with the migrant families. The third seminar was offered once with an attendance rate of 3%. At the first seminar, the parents were informed in detail about the background and the objectives of the study, as well as the measurements and the details of the intervention. Furthermore, they were informed about the importance of physical activity and motor skills for a healthy development of the children and the importance of being a role model to their child with respect to a healthy and active life style. During the second evening, a team of a dietician, two physical education scientists and a physiotherapist explained the main issues around healthy nutrition in childhood including the importance of family meals. Furthermore, practical advice for individual practices was provided. The parents also received a booklet about healthy eating, physical activity and recreation in children [20]. Finally, the parents were informed about the risk of high media use in children and possible alternative recreational activities that could replace this physical inactive behaviour. The third evening was arranged in form of an expert panel (paediatrician, physical education teachers and researchers, physiotherapist, dietician) under the title "Parents ask - Experts answer".

The contents of the educational seminars were summarised in two booklets and a letter to the parents. Additionally, the parents received two further letters: The first letter presented possibilities of an active lifestyle in children during the autumn and winter season. The second letter contained further details and child-appropriate options for a healthy nutrition. Finally, parents of the children in the intervention group received a general feedback after each assessment of their child regarding his or her results.

#### Training and supervision of kindergarten teachers

Kindergarten teachers completed two afternoon-workshops, one preceding the intervention and one halftime through the intervention period. In the first workshop, the kindergarten teachers were familiarised with the aims and the background of the study. They received information about the relevance of physical activity and motor abilities in children, healthy eating and the psychomotor approach to early childhood education. By putting theory into practice, they became acquainted with the various contents of the intervention programme such as the collection of games and exercise tasks, the daily physical activity lessons and the activity homework cards. Furthermore, the first workshop contained information on the evaluation tools of the project including the different questionnaires and the measurement techniques used.

The second workshop elaborated on the importance of physical activity during childhood and the possibilities to enhance it using the psychomotor approach. Furthermore, the second workshop addressed healthy eating strategies and motor development of preschool children. During the intervention period, there were regular exchanges between the kindergarten teachers and the research team. Supervisions that supported the individual work of the kindergarten teachers took place regularly every two weeks for at least one activity lesson in the kindergartens. Upon request, supervision visits in addition to those routinely scheduled were realised.

### Main outcomes

#### Primary outcomes

- Increase in percent time spent in moderate-and-vigorous physical activity from baseline to half-time and end of the intervention (assessed by accelerometry)

- Increase in a composite score of motor skills from baseline to two time points during the intervention: half-time of the intervention and end of the intervention (obstacle course, one foot stand, standing long jump, balancing on one foot, jumping to and fro sidewise)

#### Secondary outcomes

- Increase in percent time spent in moderate-and-vigorous physical activity between baseline and at the follow-up 2-4 months after the intervention (assessed by accelerometry)

- Increase in a composite score of motor skills between baseline and the follow-up 2-4 months after the intervention (obstacle course, one foot stand, standing long jump, balancing on one foot, jumping to and fro sidewise)

- Decrease in body mass index

- Decrease in skin fold thickness

- Decrease in blood pressure

- Increase in complex motor performance (assessed by obstacle course)

- Increase in balancing skills (assessed by one foot stand, balancing backwards, ground-reaction force platform)

- Increase in jumping skills (assessed by standing long jump and jumping to and fro sidewise)

- Increase in flexibility (assessed by stand and reach)

- Increase in throwing skills (assessed by target throw)

- Decrease in frequency of accidents

- Decrease in frequency of infections

- Decrease in media use

### Evaluation

The intervention will be evaluated for the primary and secondary outcome measures as described above. Furthermore, a process and result-oriented evaluation will be conducted based on standardised questionnaires for the kindergarten teachers and the parents of the children participating in the intervention based on questionnaires given to the kindergarten teachers and to the parents at the end of the intervention. In addition, an unstructured interview was conducted with the kindergarten teachers. This allows a closer view of the feasibility, appreciation and the parents' and teachers' appraisal of the programme's effectiveness.

### Assessments

Measurements (see Additional file 1 for overview) took place at baseline (May to July 2007), at a first follow-up about halftime through the intervention period (December to February 2007/2008), at the end of the intervention (May to July 2008), and at the follow-up about 2-4 months after the end of the intervention (September to November 2008). In general, the measurements were taken by the same research team. However there was a change in some of the investigators between the second and the third assessment periods. The assistance staff was trained as a group prior to each period to minimise inter-observer variability and was blinded to the children's allocation. In case of absence of a child another testing appointment was scheduled some days or a few weeks later. With each assessment, the parents completed questionnaires as summarised in Additional file 2 and described in more detail below.

#### Physical activity

Free living physical activity was assessed by accelerometry (GT1 M, ActiGraph LCC, Pensacola, US) during each assessment period. The children wore the accelerometer with an elastic waistband on the hip over one week at baseline (summertime), at the follow-up test 4-6 months later (wintertime), at the second follow-up test 10-12 months after the baseline assessment (summertime), and at the third follow-up test 2-4 months thereafter (autumn). The ActiGraph (formerly CSA/MTI) is the most commonly used accelerometer in physical activity research [21] and meanwhile well evaluated for the use in preschool children [22]. The parents kept an "accelerometer diary" to report when and why their child had taken off the accelerometer. In case of technical failure, illness or lack of data, children were asked to wear an accelerometer for an additional week. Epoch time was set to 15 seconds. Night time was defined between 9.00 p.m. and 6.59 a.m. and was excluded from analysis. Periods of ten minutes or more of continuous zero counts were classified as "non worn time" and also excluded from analysis. Only data from children who wore the accelerometer for at least 7 hours per day on at least 3 valid weekdays and 1 valid weekend day were included in the analysis. Time spent in moderate-to-vigorous physical activity was calculated by using a cut off of 420 counts/15 seconds [22]. Data on time spent in moderate-and-vigorous physical activity (MVPA) was calculated as average that was related to individual wearing time per day.

#### Obstacle course

As described by Kunz [3], each child ran from a marking cone to a transversally positioned bench (distance: 1 m), climbed over it, turned around, crawled under the bench, ran back to the marking cone, rounded it, and passed the course two more times. Time was taken in seconds. The bench was constructed according to the German DIN 7909 standard - except for the stabilizing bar, which was left out. For transporting reasons the bench was shortened to 2 m. With a test-retest coefficient of r = 0.97 between two attempts (n = 20) within one week the obstacle course has been found to be a reliable test [3].

#### Balancing on one foot

The child was balancing on a bar of 4.5 cm width and 6.0 cm height with one foot. The choice of leg was up to the child. He or she was asked to hold the balance for 60 seconds. The free leg had to be kept in the air without touching the ground or the bar. Otherwise, time was stopped and the child was asked to move back to the correct testing position. Then, the testing time continued. During the test, an examiner counted each ground contact with the free leg as a penalty point. Total points were summed and used as score. At 30 points the test was discontinued. The test-retest correlation between two attempts within three weeks in a pilot study (n = 152) was r = 0.60 (personal communication by Klein D, Koch B, Dordel S, Strüder H, Graf C).

#### Jumping to and fro sidewise

The child was positioned on a slip-proof board (size: 60 cm × 96 cm) with a small bar (60.0 × 2.0 × 2.0 cm) of wood separating the board into two halfs according to original test instructions published for the Karlsruher Motor Screening (KMS) by Bös and colleagues [23]. The child was asked to jump sidewise over the bar with both feet as often as possible for 15 seconds. After a rest of approximately 1 minute (individual time of recovery) the task was repeated. Jump attempts with non-simultaneous foot contacts were defined as "failed". The sum of valid jumps over the two 15-second-periods was taken. The test-retest reliability coefficient for an 8-day interval between tests lies between 0.80 and 0.90 [23].

#### Standing long jump

The child was positioned with both feet behind a line according to the KMS-test instructions [23]. The child was asked to jump as far as possible, taking off with both feet. Bending the knees and swinging the arms was allowed. The jumped distance was measured with a fixed tape on the floor and the zero point at the starting line. The child had two attempts. If the child fell backwards on its hands during or after landing, this jump was taken as an executed but failed attempt. The best valid distance of the two attempts was taken. The test-retest reliability coefficient for the standing long jump is greater than 0.80 [23,24].

#### Anthropometry and body composition

Height and weight of the children were measured to the nearest 0.1 cm and 0.1 kg in light clothing and without shoes. Skin fold thickness was determined by Holtain calliper (HSK-BI, British Indicators, UK). While the children remained standing, measurements were obtained on the right side of the body at two sites on the arm - over the triceps (midpoint of the acromion and olecranon processes on the posterior aspect of the arm) and over the biceps (midpoint of the muscle belly) - and two points on the trunk - subscapular (inferior angle of the scapula) and supra-iliac (oblique fold on the iliac crest in the mid-axillary line). The median of three measurements on each location was taken and the sum of four skin folds as well as the triceps skin fold thickness were used for analysis.

#### Blood pressure and pulse

After five minutes of rest and before starting with any of the exercise tasks, the children's blood pressure and pulse rate was measured on the right arm in triplicate with an oscillometric system (Dinamap 8100, Critikon) while sitting. Although blood pressure values measured with such a system are not concordant with results of an auscultatory system, there are good reasons for using oscillometric systems in children [25]. With very few exceptions the same investigator took these measurements. The mean of the first two readings was taken to represent the children's systolic and diastolic blood pressure. If the difference between the first and the second reading was >5 mm Hg, a third reading was used in addition to the first two measurements to calculate the mean [26].

#### Balancing backwards

According to the manual of the MOT 4-6, a motor test battery for preschool children [27], the child was asked to balance backwards on a carpet strip that was 10 cm wide and 2 m long without stepping off the strip. The way the child approached the task was optional (sliding the feet, making small or large steps). This task was repeated once. One examiner positioned behind the child judged the success (no steps apart) or failure (steps in part or completely off the carpet strip). Two successful attempts were noted as 2 points, one successful attempt was recorded as 1 point and 0 points were noted for no successful attempt.

#### Target throw

Corresponding to the instructions of the MOT 4-6 [27], the child tried to hit a round target 40 cm in diameter, fixed on the wall in 3 m distance and at the height of 1.70 m (upper border), with a conventional tennis ball. Each child had four attempts including one practice attempt. The examiner standing behind the child decided about successful hits of the target - defined as fully or partly touching the disk - and documented the results (no hit: 0 points; 1 hit: 1 point; 2 and more hits: 2 points).

#### Stand and reach

As described by Bös and colleagues [23], the child stood on a wooden box of 30 cm height, 40 cm width and 31 cm depth behind a wooden board. The child was asked to bend over without flexing the knees and to reach down as far as possible with the fingers. When the child had problems to stretch the knees, a second examiner gave support. The distance between the fingertips and the platform level was measured in centimetres. If the finger tips remained above the platform the distance was recorded in negative numbers, if they reached below platform as positive number. The test-retest reliability coefficient has been reported to range between 0.80 and 0.90 [23].

#### Static balance on a force platform

A ground-reaction force platform (*Balance-X-Sensor*^®^, *Soehnle Professional GmbH, Murrhardt, Germany*) was used for assessment of static postural balance. The device is based on three piezoelectric weighing cells located in a triangle with a sensitivity of ±1 N and a maximum load of 4 kN per cell. This platform is transferring muscle forces acting against gravity as total force and in x-y coordinates. The detection of forces within the spanning triangle is ±1N. The centre of gravity is located with an accuracy of ±1 cm at 20N. Mechanical waves produced by forces against gravity on the platform are recorded as function of time f(t) at a sampling rate of 100 Hz. The testing allows an analysis of the power spectral density distribution (PSDD) in Watt per Hertz, the average mechanical power afforded during the test as an integral of PSDD, the trace length of centre of mass displacement (mm) and the force vector area of the displacements (cm^2^). Details of the technical system are described elsewhere [28].

After calibrating the system for the specific weight of the subject, the child was placed barefoot on the platform with one foot in front of the other, balancing on a marked line with open eyes. The heel of the fore foot touched the toes of the rear foot. Arms had to be kept close to the body. The child was asked to keep this position with as little movements as possible. Test time was set at 10 seconds. After a test-stand on the platform, each child had three attempts. The best valid attempt was taken for analysis.

#### Migrant status and socio-economic status

The children's parents were asked to fill in questionnaires during each testing period (see Additional file 2 for content). Since there were families with a Russian or Turkish background and poor German skills, questionnaires were translated into these languages to ensure a valid data acquisition from the respective parents.

The assessment of migrant status was conducted according to the standard of the Kinder- und Jugendgesundheitssurvey (KiGGS), a nation wide representative survey assessing the health status of German children. Thus, a child is considered as migrant if: 1) The child has immigrated from another country and one parent was not born in Germany, or 2) both parents have immigrated to Germany or do not have the German citizenship [29].

To describe the socio-economic status (SES) the Winkler index was used [30]. For dividing experimental subjects into those with low, middle or high SES, this index uses three characteristics of each person (education, represented by the school and professional education; actual or recent professional position; net household income). In each of these three areas a score between 1 and 7 is given. Then, the sum of the three scores is calculated. Low SES is defined reaching a score ≤8, middle SES between 9 and 14 scores and high SES ≥15. If SES of both parental persons was available and the child was living with both parents, the higher score was taken to denote the SES of the child. If the child lived with either mother or father the respective parental score marked the child's SES.

#### Health related data

Parents gave information about the general health status of their child concerning the existence of chronic health problems, i.e. pulmonary, cardiac, and orthopaedic diseases. Furthermore, they answered questions about the following health-related issues: recent hospitalisations of the child, use of medication, and participation in physiotherapy or ergotherapy. This information was used only to identify children with serious chronic health problems who were excluded from the study.

Accidents and infections that were accompanied by a reduction in physical activity of the child for at least 6 hours and/or required consulting a doctor were reported by the parents' retrospectively for the previous three months (accidents) and for the previous four weeks (infections), respectively. Parents were further asked to provide information on the kind of accident and/or infection, the amount of hours with reduced physical activity due to the accident/infection of the child and, in case of reported accidents, on the kind of injury.

The average duration of the child's sleep was addressed in the questionnaire separately for weekdays and weekend.

Furthermore, the parents were asked to fill in their present height (cm) and weight (kg).

#### Leisure time activities

Media use was assessed as the daily and weekly amount of television viewing, watching videos and playing computer games in minutes, reported by the parents. Furthermore, the parents gave information on the frequency of the child's television viewing per week ("every day of the week", "for 4-6 days a week", "for 1-3 days a week", "less than 1 day a week or never").

The physical activity of the child, of the mother and of the father was assessed by questions asking for sports participation in a club and the time spent in non-organised sports. The information requested included the kind of sports participation, the frequency per week or per month and the duration per workout (for example: soccer 2 × 1 hour per week, cycling 7 × 10 min per week).

The questionnaire also asked for information about the frequency the child played outdoors ("every day of the week", "for 4-6 days a week", "for 1-3 days a week" or "seldom"). Times of outdoor play of the child with and without the mother or father were separately assessed. The parents also indicated the amount of time they spent with the child during outdoor play per week. Additionally, the parents were asked to estimate the intensity of their child's activity outdoors and also their own exertion during the joint playing time separately for each parent ("sweating and getting breathless?", answers "yes" or "no").

#### Parental evaluation of the programme and monitoring of attendance for children in the intervention group

The parents of the children in the intervention group were asked twice - half-time through the intervention and at the end of the intervention - to report their satisfaction with the organisation of the project, as well as their evaluation of the physical activity homework cards and the collection of games and exercise tasks. Furthermore, the parents appraised the children's acceptance of the programme and the effects of the intervention they might have noticed in their child. Changes in the child's characteristics specifically addressed by questions including activity level, motor skills, accident frequency, concentration, temper, and general health were documented by the parents. For each of these characteristics, a classification on a 6-point Likert-scale was requested, with additional space for comments.

The kindergarten teachers were asked to keep an attendance record to document the participation of the children in the intervention lessons.

### Data analysis

A composite motor skills score will be calculated by averaging the z-transformed results of the obstacle course, the standing long jump, the balancing on one foot task, and the jumping to and fro sidewise task to describe the baseline situation. Likewise, the changes in performance from baseline of the above motor skills tests will be z-transformed and averaged for each of the three follow-up time points. For these computations, the values of the obstacle course and the balancing task will be multiplied by "-1" to account for the fact that a low score in these tasks indicates a better performance.

Baseline data will be explored by descriptive statistics. Differences between the intervention and the control group at baseline will be analysed by two sample Students t-tests for normal distributed interval scaled variables, by Wilcoxon Tests ordinal and/or not normal distributed variables and by Chi-squared statistics for nominal variables.

The primary endpoints will be analysed by a repeated measurement analysis of covariance with the changes form baseline to the two assessments during the intervention period as dependent variables and including the fixed factors intervention, gender, and urban/rural location of the kindergarten, the random factor kindergarten and the covariate age in the model. For the composite score, the mean of the z-scores of the changes in performance and the mean of the z-scores of the baseline values are used respectively. The same statistical procedure will be used for the secondary outcome variables in an exploratory manner. Predefined gender specific subgroup analyses will be performed. Several additional explorative analyses will also be performed concerning the intervention effects on selected primary and secondary outcomes in specific subgroups, such as children with different body composition, children with low motor skills performance at baseline, children with low socioeconomic status and or with a migrant status, respectively.

As effects of the intervention are anticipated to become apparent in the entire distribution of motor skills scores, no specific threshold is presumed.

There are two primary endpoints, namely the change in moderate-and-vigorous physical activity and the composite score of changes in motor skills during the intervention period. Therefore, the Bonferroni adjusted 2 sided significance levels of 0.025 are used for the analysis of primary endpoints. For all secondary endpoints, statistical significance will be considered at p < 0.05.

Effects will be analysed primarily as intention-to-treat-analyses and additionally as per-protocol analyses.

## Discussion

The purpose of our study is to proof the feasibility and effectiveness of a multilevel intervention in preschool children aiming to increase physical activity and motor skills. The effects on adiposity, media time, blood pressure, infections and accidents will also be assessed.

We believe that the involvement of the kindergarten teachers in the activity programme will not only translate in an effective intervention tailored to the need of each individual facility, but also in a continuation of the programme after the end of the formal intervention period. Furthermore, it is likely that the empowerment achieved by this study in the kindergarten teachers might also facilitate the transfer to other kindergartens since an involvement of the intervention kindergartens in the training of other teachers is planned. If the study can show feasibility and effectiveness of the intervention, the nationwide transfer of the project will be realised in cooperation with a health insurance company (BARMER GEK).

## Competing interests

The authors declare that they have no competing interests.

## Authors' contributions

HH, CG, KR, and WL were responsible for the design of the study with HH as principal investigator and guarantor. HH and KR were the main coordinators of the study. HH, CG, KR und KCR established the methods and the questionnaires. HH, KR, KCR, SM and MO conducted the study. KR, KCR and SM developed the curriculum of the programme with its practical and informational components. DL developed the nutritional part of the intervention. WL did the power calculation for this study and is counselling the statistical analysis. HH obtained the funding. KR drafted the paper with SK, HH and KCR critically revising it. All authors have read and approved the final version.

## Pre-publication history

The pre-publication history for this paper can be accessed here:

http://www.biomedcentral.com/1471-2458/10/410/prepub

## Supplementary Material

Additional file 1 **Measurements taken in the PAKT-Study**. Click here for file

Additional file 2 **Content of questionnaires given to families and kindergarten teachers**. Click here for file
