# Supplementary material for: Prevention through Activity in Kindergarten Trial (PAKT): A cluster randomised controlled trial to assess the effects of an activity intervention in preschool children
Source: BMC Public Health. 2010 Jul 12;10:410. doi: 10.1186/1471-2458-10-410 (PMC2916900; doi:10.1186/1471-2458-10-410)
Supplement: Additional file 1 — Measurements taken in the PAKT-Study. [file 1471-2458-10-410-S1.PDF]

Table 1: Measurements taken in the PAKT-Study

Physical activity

- Accelerometery (GT1M, ActiGraph) (Moderate-and-vigorous physical activity, % of total recording time)
- Questionnaires

Motor skills (Sum Score)

- Obstacle course (body agility, coordination and speed) (s)
- Balancing on one foot (stabile balance ability) (ground contacts with the free foot in 60 s)
- Jumping to and fro sidewise (coordination and short-term power) (jumps in 2*15 s)
- Standing long jump (springiness) (cm)

Motor skills (single item analysis)

- Balancing backwards (labile balance ability) (successful attempts)
- Target throwing (coordination and throwing precision) (successful attempts)
- Stand and reach (flexibility) (cm)
- Static balancing (ground reaction force platform) (power spectral density distribution, mechanical power, trace length of centre of mass displacement, force vector area of the displacements)

Anthropometry

- Height (cm)
- Weight (kg)
- Skinfold thickness (triceps, biceps, subscapular, suprailiacal) (mm)

Blood pressure

- Systolic and diastolic (mm Hg)
