# Supplementary material for: Prevention through Activity in Kindergarten Trial (PAKT): A cluster randomised controlled trial to assess the effects of an activity intervention in preschool children
Source: BMC Public Health. 2010 Jul 12;10:410. doi: 10.1186/1471-2458-10-410 (PMC2916900; doi:10.1186/1471-2458-10-410)
Supplement: Additional file 2 — Content of questionnaires given to families and kindergarten teachers. [file 1471-2458-10-410-S2.PDF]

Table 2: Content of questionnaires given to families and kindergarten teachers

Questionnaires for the parents

- Socio-economic status of the family
- Migrant status of the child
- General health of the children
- Accidents
- Infections
- Physical activity
- Time outdoors of the child and the parents
- Sports club participation of the child and the parents
- Media use of the child
- Sleep duration of the child
- Height and weight of the parents
- Evaluation of the intervention*

Questionnaires for the kindergarten teachers

- Evaluation of the intervention*

* these questionnaires were given only to the parents and teachers of the intervention group
